# The History, Pathology, and Treatment of Puerperal Fever, and Crural Phlebitis

**Published:** 1842-08-20

**Authors:** 


### MEDICAL EXAMINER.

NEW SERIES.

No. 34.] PHILADELPHIA, AUGUST 20, 1842. [Vol. I.

### BIBLIOGRAPHICAL NOTICE.

*The History, Pathology, and Treatment of Puerperal Fever, and Crural Phlebitis.* 1. *A Treatise on the Epidemic Puerperal Fever of Aberdeen.* By ALEXANDER GORDON, M. D. 2. *A Treatise on the Puerperal Fever, &c.* By WILLIAM HEY, Esq. 3. *Facts and Observations relative to the Fever commonly called Puerperal.* By JOHN ARMSTRONG, M. D. 4. *On Puerperal Fever and Crural Phlebitis.* By ROBERT LEE, M. D., F. R. S. *With an Introductory Essay, by* CHARLES D. MEIGS, M.D., Professor of Obstetrics and Diseases of Women and Children in the Jefferson Medical College. Philadelphia: Ed. Barrington & Geo. D. Haswell. 1842.

For this collection of valuable monographs on the subject of puerperal fever—some of them out of print—we are indebted to Professor MEIGS, who adds an interesting introductory essay. The work of Dr. Gordon, the first of the series, (London, 1795,) was the earliest to present sound views of the therapeutics and pathology of this fearful disease. It is a history of a severe epidemic of puerperal fever which prevailed at and near Aberdeen, in Scotland, from December, 1789, to March, 1792. The first cases which came under the Doctor's care were treated "without energy and without success." He was then led to adopt a more vigorous practice, consisting of free bleeding from the arm, aided by the use of purgative medicines, which resulted in the recovery of nearly two-thirds of his patients. The publication of Gordon, "the reformer of the therapia of puerperal fever," as Dr. Meigs styles him, has been long out of print. The profession of this country are under obligations to Dr. Meigs for placing within their reach, "at a cheap rate," this plain, graphic, and evidently candid detail of a formidable epidemic.

Dr. Hey's treatise contains a collection of well drawn up cases, illustrative of the necessity and good effects of a vigorous antiphlogistic treatment. Together with these works, Armstrong's "Facts and Observations," and the ample and elaborate pathological details of Robert Lee, constitute what the editor aptly denominates "a beautiful series, rising from a simple proclamation of a deeply interesting and important principle, in the history of the Aberdeen fever, to an illustration and enforcement of it in the Leeds and Sunderland writers; while a most full, elaborate, and convincing display of the whole subject is found in the London author. The progression is complete."

Dr. Meigs has, during the course of a very extensive practice, resorted to "a vigorous employment of the lancet" in the management of metro-peritonitis. In this Introductory Essay, he warmly inculcates this course of treatment. He moreover strongly insists upon the superiority of general to local bleeding, regarding the latter as inadequate to the intense and rapidly advancing inflammatory symptoms. He perseveres with bleeding from the arm, at all hazards, until an impression be made on the pulse, and cites cases in which, in spite of enormous quantities of blood previously lost by flooding, repeated and free blood-letting was successfully resorted to. Of the importance of free bleeding *from the arm* in inflammations of serous membranes generally, there can hardly be a doubt; and in the violent and wide-spread inflammation which accompanies metro-peritonitis, it is probably our main reliance. We extract the following cases from Professor Meigs' Essay, in both of which a decided practice was attended with the best results.

"A young married lady, at the end of her fourth pregnancy, was attacked about 3 A. M. with pains of labor and flooding. I did not see her until midday. Upon arriving at the house, I judged, upon careful inspection of the napkins and sheets, &c., that had been removed from about her person, that she had lost fifty ounces of blood; an opinion strengthened by an examination of her pulse, her muscular strength, and her skin, which was excessively pale. Upon making an examination I found the hemorrhage still active; and, in order to check it, resolved, as the placenta was not in reach, and the os uteri one-third dilated, to rupture the membranes, according to the method, as it is called, of Louise Bourgeois. As soon as the water had gone off, the hemorrhage was stayed; and she not long afterwards gave birth to a healthy child. The young lady was very weak. I saw her late in the evening, and she was comfortable, but extremely pale. During the course of the next day and night she was comfortable, and was kept carefully in bed, being without other complaint than debility. But at 4 o'clock in the morning she was attacked with intense rigor, amounting to ague, accompanied by excessive pain and soreness of the belly, with a pulse at 150 per minute, rising at times to 160 beats; and she presented indeed, all the phenomena of a violent attack of puerperal fever. I did not see this patient until as late as 11 A. M.

Upon making out the diagnosis, I again made inquiries of her mother, a most intelligent person, who convinced me that the first estimate as to the quantity lost in the hemorrhage was far too small; and I have not, at this moment, any doubt of her having lost on that day full seventy ounces of blood. As puerperal fever was prevailing considerably at that time, I felt deeply concerned as to the line of my duty in the actual circumstances. It appeared to me more than probable, considering the violence of the attack after so great a hemorrhage, that it would prove fatal under any treatment that might be adopted. I thought it certainly would have a fatal result, should I allow the heart to continue beating at so violent a rate. 150 pulsations per minute will give 115,200 pulsations per diem over and above the number required for the healthful rate of the circulation; which, at 70 per minute, gives a little over 100,000 per day. Let any one conceive the amount of danger and mischief concomitant with the long continuance of such an excessive rate of vital function. But, the patient had already lost profusely of her blood; and hence, with due regard to my own reputation, or the credit of a most invaluable remedy, could I venture to increase this loss by bleeding her, whose death, probable under any treatment, would be boldly charged to mal-practice. It was a severe struggle; but I had firmness enough to follow the suggestions of my judgment; and having bled her as freely as I dared to do, I had the satisfaction to find that the pulse soon fell to a more moderate state, and in forty-eight hours my patient was out of danger, and is now enjoying health, and a life which I sincerely think would have been destroyed by metro-peritonitis, but for the correct decision I made as to the use of the lancet."

"Mrs. W. G., aged about twenty years; first pregnancy; was delivered on the fourth of November, Thursday, 1830, of a female child after a labour of four hours.

"She was very comfortable on Friday and Saturday. There was already a small quantity of milk in the breasts, but they were neither full nor painful. The bowels were opened on Saturday by a dose of castor oil, a table-spoonful and a half, which operated through the day and night ten or twelve times. She has had, for her diet, tea and bread, and oatmeal gruel. There was no fever on Friday or Saturday.

"*Sunday*, November 7th.—I did not call to see her until past ten A. M. She had had a chill in the night, rested badly, and now suffers pain and soreness in the right flank and iliac region. The parts were very tender on pressure, distended and resonant under percussion; the *fundus uteri* above the symphysis pubis sore to the touch; lochia bright and free; urine abundant; tongue whitish, soft, moist, and broad; headach; thirst; dorsal decubitus; motion of thighs gives pain in abdomen; any attempt to rise or turn also gives pain; pulse 148, with a vigorous stroke. She was bled 18 ounces from a large orifice, when faintness came on and the arm was bound up. In a few minutes after the bleeding the pulse was 112, but it soon rose again to 152. Although the bowels had been moved so often, I thought their flatulent state indicated an arrest of the peristaltic movement from inflammation of their peritoneal coat; and to re-excite them, she took a common enema, which operated twice with relief. A flannel bag, filled with wheat bran soaked in boiling vinegar and water, after being well pressed, was laid warm on the belly: it was changed occasionally.

"At twenty minutes past 3 P. M. The pulse 145, with a smart stroke; the tenderness of the belly neither less nor greater. I took 12 ounces of blood from the arm in a large stream. I was obliged to stop on account of faintness, though I had first drawn the pillows from under the head in hopes of getting a larger quantity. In fifteen minutes afterwards pulse 144.

"At 4½ P. M. Calomel, gr. viij.; Opium, gr. iss. The powder was taken for a dose. To drink gum water.

"At 6½ P. M. Has slept, and feels decidedly less pain and soreness; but as the pulse is frequent and strong, I took twenty-two fluid ounces of blood, which was carefully measured. It made a firm clot, and had a thick coat of size. She took Manna, ℥ss.: Sem. anise, ʒi.; Magnes. carb. ℥ss.; Aq. bullient. ℥vi. An infusion was made of the anise and manna in the boiling water; which, when cool, was strained: after which the magnesia was added to make a proper mixture. A fluid ounce was taken for a dose every hour until the bowels were moved.

"At $10\frac{1}{2}$ P. M. Pulse 136, full and strong; the right mamma filling and hardening, the left soft and flaccid, but the gland is developing favorably; no headache; thirst; the soreness and pain on pressure (carefully examined) are very much lessened; lochia free; *decubitus* still dorsal.

"*Monday*, 8 A. M. Has slept a good deal; pulse 130, and softer; no pain, except by firm pressure on abdomen; thirst lessened; both mammæ full and hard.

"3 P. M. Pulse 120, full and strong; no pain, not even from pressure; tongue clean; had several stools; not thirsty.

"$9\frac{1}{2}$. Same.

"*Tuesday*, 9th November, 9 A. M. Pulse 124, and strong; tongue somewhat furred; plenty of milk; breasts sore; no pain; bowels moved again.

"9 P. M. Pulse 111; sore nipples.

"*Wednesday*, 10 A. M. Has been sitting up; no pain; pulse 126.

"In a few days after this last date she was perfectly well.

"This young woman had a healthy and strong constitution. In her case I took away, between eleven and six o'clock on the first day of the attack, fifty-two ounces of blood, without which, I think, she must have died.

"I have related the above case from my note book. I present it as a fair specimen of the mode of practice, in such attacks, which I have for years been in the habit of pursuing. I have treated cases in the Pennsylvania Hospital and in private houses upon the same principle; and I have the satisfaction to say, that my just expectations of success, founded on the doctrines of Gordon, have rarely been disappointed."

